# New Modified SPEEK-Based Proton Exchange Membranes

**DOI:** 10.3390/polym17121646

**Published:** 2025-06-13

**Authors:** Fátima C. Teixeira, António P. S. Teixeira, Carmen M. Rangel

**Affiliations:** 1 Laboratório Nacional de Energia e Geologia, I.P., Estrada do Paço do Lumiar, 22, 1649-038 Lisboa, Portugal; carmen.rangel@lneg.pt; 2 Departamento de Ciências Médicas e da Saúde, Escola de Saúde e Desenvolvimento Humano & LAQV-REQUIMTE, Instituto de Investigação e Formação Avançada, Universidade de Évora, R. Romão Ramalho, 59, 7000-671 Évora, Portugal; apsteix@uevora.pt

**Keywords:** SPEEK modified membranes, proton exchange membranes, fuel cells, electrolyzer

## Abstract

A decarbonized society demands cleaner and sustainable energy sources based on well-established or emerging technologies with the potential to make a significant contribution to energy storage and conversion, such as batteries, fuel cells and water and/or CO_2_ electrolyzers. The performance of these electrochemical devices relies on key components such as their separators/ion-exchange membranes. The most common commercial membrane, Nafion^®^, has several technological limitations. In this study, it is proposed the incorporation of bisphosphonic acid (BP) dopants into membrane matrices to improve their properties. Following this strategy, we prepared new membranes based on sulfonated poly(etheretherketone) (SPEEK) polymer, a reliable and effective alternative membrane polymer, through the incorporation of the BP dopants, to obtain low-cost membranes with improved properties. These membranes were structural, thermal and morphological, characterized by AT-FTIR, TGA and SEM. Their proton conductivity was evaluated over a temperature range between 30 °C and 60 °C, using Electrochemical Impedance Spectroscopy, and their stability during this process was also observed. The best proton conductivity was observed for the SPEEK membrane doped with **BP1** at 2.0 wt% load at 60 °C, with a proton conduction of 226 mS cm^−1^.

## 1. Introduction

A demanding net-zero emission decarbonized society requires clean, sustainable and environmentally friendly energy sources that can be alternatives to traditional fossil fuels [1,2]. The implementation of ambitious plans for national and international decarbonized energy systems requires the production, conversion and storage of clean energy, including stable, with continuous sources and permanent intensity, that enables a full supply for the needs of society activities [3]. Renewable energy sources, such as wind and solar, are very dependent on the meteorological conditions and periods of the day, remaining intermittent providers, unable to ensure the full needs of energy at all times [4]. This can be complemented and ensured by power systems involving electrochemical devices capable of permanently supplying the necessary energy [5,6,7]. From well-established to emerging technologies, from energy conversion to energy storage, electrochemical devices, such as batteries, fuel cells or electrolyzers, can make a significant contribution to this 21st-century societal endeavour [8,9,10].

However, electrochemical devices still rely on key components to ensure their quality and technological applications. Among them, membranes remain a critical component of these devices, acting as a separator, promoting an efficient separation of all reactants and products of the reaction process, preventing an uncontrolled flow of materials or electrolytes while permitting the proton conduction between the electrodes of these devices, tuning the crossing of the selected species. These membranes can have a greater diversity of technological applications, but the performance of their devices depends crucially on the composition of the proton exchange membranes [11,12,13,14].

Membranes have been prepared from various polymers, set from various functionalized organic monomers, many of them presenting different degrees of fluorination [15]. Among the membranes, the perfluorinated sulfonic Nafion^®^ membrane stands out, which, despite its cost, has the greatest commercial success [16,17]. Nafion^®^ is the most widely used commercial membrane but has several limitations, including its limited operating temperature range due to the dependence of its performance on water content. The incorporation of dopants into its polymeric matrix has shown that the strategy can surpass some of its limitations [18].

The limitations of actual membrane matrices and their importance on the diverse electrochemical devices still foster the investigation and development of new alternative membranes [19,20]. Poly(etheretherketone) (PEEK) polymer is a versatile non-fluorinated material that can be functionalized to the introduction of sulfonic acid groups to obtain a sulfonated poly(etheretherketone) (SPEEK) material [21,22,23]. This SPEEK polymer can be easily cast to achieve a low-cost and environmentally friendly membrane with excellent thermal and chemical stability and higher proton conductivity [24,25]. These properties can be partially tuned by the use of SPEEK polymers with different degrees of sulfonation (DS) (related to the quantity of sulfonic acid groups present at the polymer unit), which is obtained according to the experimental conditions used during the sulfonation conditions [26,27]. Usually, a higher degree of sulfonation promotes proton conductivity and improved mechanical stability and resistance to acidic conditions but decreases the performance and stability while leading to swelling [28].

Previous studies have shown that the incorporation of phosphonic acid dopants into Nafion^®^ membranes can modify their properties, including the improvement of their conductivity and stability, due to the participation of the acid groups of the dopants in the modification of the proton conduction [29,30,31,32]. It was also observed that the presence of phosphonic acid groups at the membrane could result in enhanced proton conduction due to a lower energy barrier (37.2 kJ mol^−1^) compared to sulfonic acid groups (69.9 kJ mol^−1^) [33,34], which can be reinforced by the self-ionization of phosphonic acid groups resulting on a dual behaviour as proton donor and conductor [35,36,37].

Considering the properties of both SPEEK membranes and phosphonic acid dopants, it is anticipated that new SPEEK membranes prepared through the incorporation of these dopants can result in better proton conductivity membranes. In this work, we report the preparation of new membranes based on sulfonated poly(etheretherketone) (SPEEK) polymer, a reliable and effective alternative to Nafion, through the incorporation of the bisphosphonic acid (BP) dopants to obtain low-cost membranes with improved properties. This study also allowed the characterization of the new membranes and the evaluation of their proton conduction.

## 2. Materials and Methods

### 2.1. Materials and Chemicals

Poly(oxy-1,4-phenyleneoxy-1,4-phenylenecarbonyl-1,4-phenylene) (Poly(etheretherketone)-PEEK) powder (mean particle size 80 μm) and other reagents were acquired from Sigma-Aldrich and used as received.

### 2.2. Preparation of Dopants

**Figure 1:**
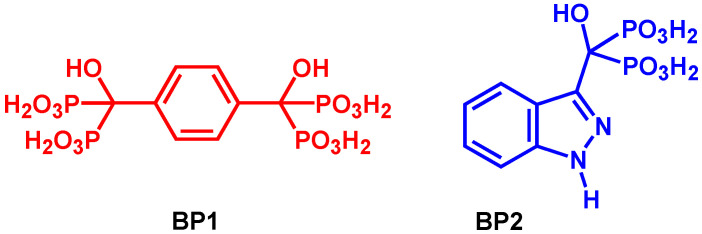
Structure of **BP1** and **BP2** dopants.

Dopants [1,4-phenylenebis(hydroxymethanetriyl)]tetrakis(phosphonic acid) (**BP1**) and [hydroxy(1H-indazol-3-yl)methanediyl]bis(phosphonic acid) (**BP2**) were synthesized in conditions already established within the group [38,39]. Their chemical structures are shown in Figure 1.

### 2.3. Preparation of SPEEK

**Figure 2:**
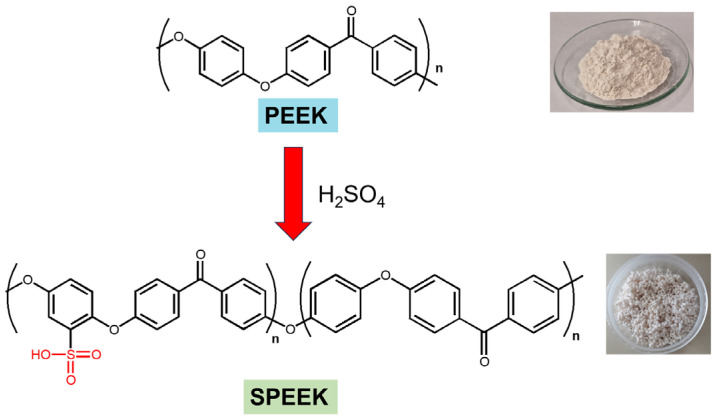
Preparation of SPEEK at room temperature.

SPEEK polymer was prepared by sulfonation of PEEK with sulfuric acid, and its degree of sulfonation was determined by ^1^H NMR. PEEK particles were dried at 100 °C overnight. Dried PEEK (2 g) was progressively added to concentrated sulfuric acid (40 mL). The mixture was vigorously stirred for 5 days at room temperature under a nitrogen atmosphere. Then, the solution was added slowly to ice-cold water to precipitate the polymer. The mixture was stirred for 2 h and left to rest for 24 h. After filtration, the solid SPEEK polymer was washed with deionized water several times until neutral pH solutions were discarded. The resulting solid SPEEK was then dried at room temperature and at 80 °C for 24 h (Figure 2).

PEEK sulfonation was also carried out over a long stirring period of time (12 days) using the same methodology.

Different periods of stirring allowed the formation of SPEEK polymers with different degrees of sulfonation, which were determined by ^1^H NMR spectroscopy.

### 2.4. Preparation of Membranes

**Figure 3:**
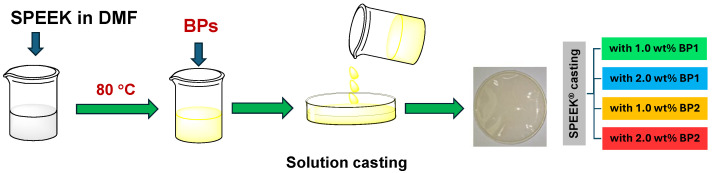
Membrane preparation.

New SPEEK membranes were prepared by a casting method (Figure 3). The SPEEK polymer was dissolved in the corresponding amount of DMF to form a 10 wt% solution of SPEEK at 80 °C for 1 h. Then, different amounts of BPs (1.0 or 2.0 wt% of **BP1** and **BP2** dopants) were added to the solution, and the mixtures were stirred at 80 °C for 1 h to ensure their complete dissolution. The obtained solutions were cast in a Petri dish, and the solvent was slowly evaporated to obtain a homogeneous membrane. The new membranes were firstly dried in a vacuum oven at 80 °C, followed by the annelation process through their heating at 100 °C for 2 h. The membranes showed an average thickness of 136 µm.

All membranes were activated by treatment with a 1.0 M sulfuric acid solution for 24 h, followed by washing with deionized water.

The prepared membranes were labelled as SPEEK/BPn-x, where n indicates the specific BP used, and x specifies the wt% of the dopant.

### 2.5. Characterization of Polymers

#### 2.5.1. Sulfonation Degree

Each sample of SPEEK polymer (10 mg) was dissolved in DMSO-*d*6 (0.5 mL), and its ^1^H NMR spectra were recorded on a Bruker Avance III HD 400 spectrometer running at 400 MHz. Chemical shifts (δ) are indicated in ppm.

#### 2.5.2. Water Uptake

Water uptake of the new membranes was determined by the weight difference between wet and dry membranes. Dry membranes were prepared by their drying at 60 °C overnight in a vacuum oven. After cooling at room temperature, their weights were measured (*W*_(*dry*)_). After their weights were recorded, they were placed in deionized water at 25 °C overnight to reach a wet equilibrium. Afterwards, the surface water was carefully blotted with absorbent paper and the membranes’ weights (*W*_(*wet*)_) were recorded.

The water uptake of membranes was calculated by Equation (1):

(1) $$Water\;uptake\;(\%)=\frac{W_{\left(wet\right)}-W_{\left(dry\right)}}{W_{\left(dry\right)}}\times 100$$

#### 2.5.3. ATR-FTIR Spectroscopy

Infrared spectroscopic analysis of the new SPEEK membranes was carried out using an ATR-FTIR spectrometer (PerkinElmer spectrometer, Spectrum Two model, with Fourier transform, with Attenuated Total Reflection (ATR-FTIR) module) over a scan range between 4000 and 400 cm^−1^. The intensity of spectra bands was recorded in % transmittance, and their wavenumbers are quoted in cm^−1^.

#### 2.5.4. Proton Conductivity

Electrochemical impedance spectroscopy (EIS) was used to evaluate the in-plane proton conductivity of the membranes. This was carried out on a Solartron 1260 frequency response analyzer coupled to a Solartron 1287 electrochemical interface between 1 MHz and 5 Hz frequencies, with a test signal amplitude of 10 mV, using a commercial BekkTech BT-112 conductivity cell, inside a temperature- and humidity-controlled Binder KBF 115 climate chamber, according to an equipment setup already described [40]. The proton conductivity was measured at 100% relative humidity (RH) conditions as a function of temperature.

Bulk resistances were estimated through the analysis of the results plotted as a Nyquist diagram by obtaining the intercept on the real axis in the high-frequency region and also by using an “a priori” equivalent circuit, including an RC arrangement, which represents the bulk and interfacial properties of the polymer. AC impedance parameters in the diagram were adjusted by a non-linear least square fitting technique using the Zview software (Version 4.0b, Scribner Associates), which allowed the determination of the bulk resistance (*R_b_*) of the membranes. The proton conductivity (*σ*) was calculated using Equation (2):

(2) $$\sigma =\frac{L}{AR_{b}}$$

where *L* is the distance between the two electrodes (cm), *R_b_* is the calculated bulk resistance (Ω) and *A* is the cross-sectional area (cm^2^) obtained by multiplication of the thickness and the width of the membrane.

#### 2.5.5. Scanning Electron Microscopy (SEM)

Surface morphology analysis of membranes was carried out by Scanning Electron Microscopy (SEM) on a Scanning Electron Microscope Philips XL30 FEG (Field Emission Gun) at a 10 kV acceleration voltage. Sample surfaces were coated with a layer of gold to enhance electrical conduction.

#### 2.5.6. Thermal Analysis

Thermal analysis of membranes was carried out by Thermogravimetric analysis (TGA) on a SETSYS Evolution 1750/Simultaneous TGA DSC Thermal Analyzer (Setaram), under nitrogen atmosphere with a gas flow of 30 mL min^−1^, at the University of Vigo, Spain (C.A.C.T.I—Unidad de Caracterización de Materiales). Samples were placed on a platinum crucible. A temperature range from 20 to 700 °C and a heating rate of 10 °C min^−1^ were used.

## 3. Results and Discussion

### 3.1. Membrane Preparation

**Figure 4:**
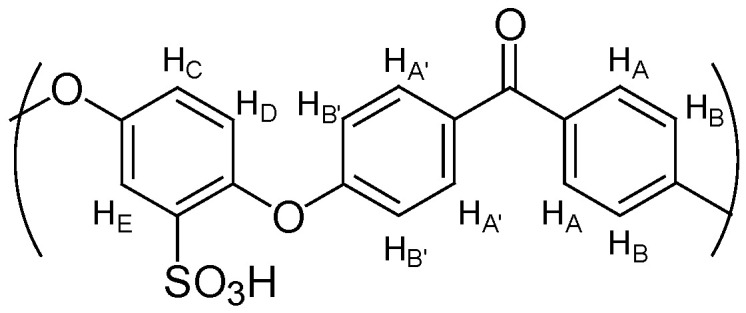
Identification of aromatic proton at the SPEEK repeated structural unit.

**Figure 5:**
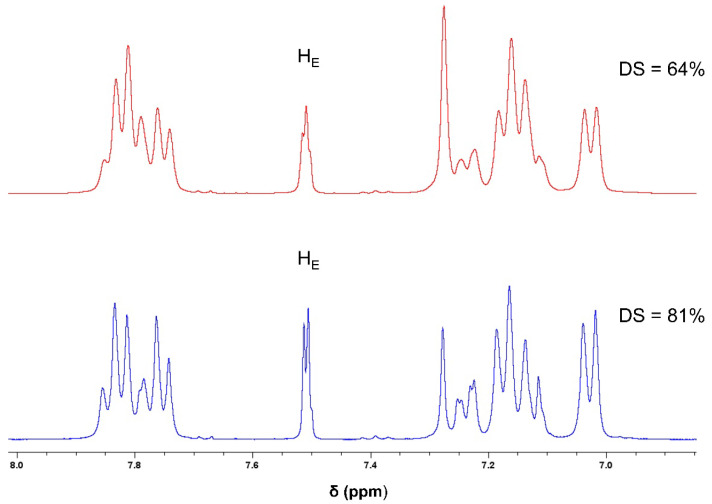
^1^H NMR spectra of SPEEK polymers with different degrees of sulfonation.

The new membranes were prepared from SPEEK polymer. This base polymer was prepared from the sulfonation reaction of PEEK with concentrated sulfuric acid at room temperature (Figure 2). Different reaction times of this sulfonation process promote different values of the degree of sulfonation (DS) [41,42]. With the sulfonation of the polymer backbone, it was observed a characteristic signal of H_E_ proton (Figure 4) at ^1^H NMR spectra (Figure 5), at deuterated DMSO solvent, with a chemical shift near 7.5 ppm. The intensity of this singlet allowed the determination of the degree of the sulfonation of the samples obtained after 5 days (DS = 64%) and 12 days (DS = 81%) reaction times, using Equation (3).

(3) $$DS=\frac{n}{12-2n}=\frac{{AH}_{E}}{{AH}_{A,A^{\prime }B,\;B^{\prime },\;C.\;D}}\times 100$$

**Figure 6:**
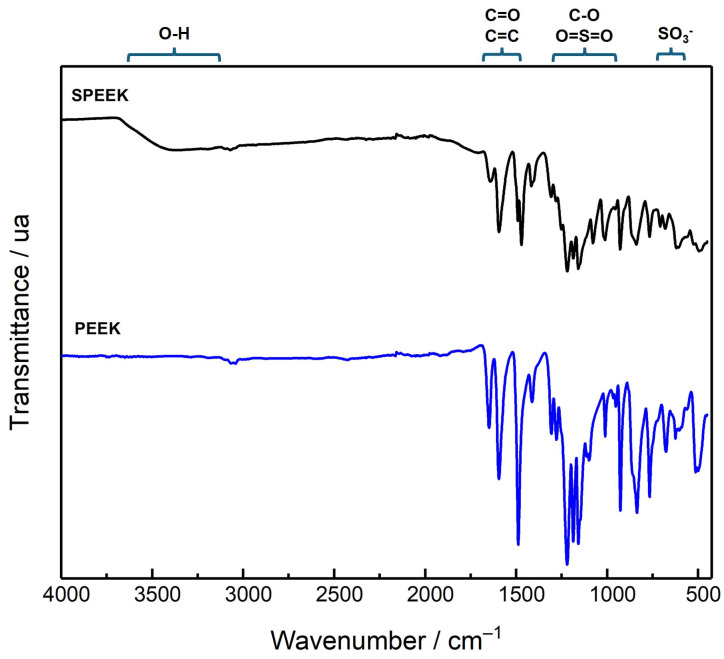
ATR-FTIR spectra of PEEK and SPEEK (DS = 64%) polymers.

Besides the different NMR spectra, the ATR-FTIR characterization of both polymers confirms the successful sulfonation of PEEK (Figure 6). The SPEEK spectra show a new and very broad band with a maximum near 3380 cm^−1^ due to the stretching of the hydroxyl O-H of the sulfonic group and the appearance of new bands around 1010–1100 cm^−1^, attributed to the symmetrical stretching of S=O bond, with the asymmetrical stretching bands near 1200 cm^−1^ being partially superimposed by C-O stretching bands. Also, the SO_3_^−^ vibrations bands can be observed at 708 cm^−1^. The stretching band of the C=O bond of the SPEEK has a slight increase in its wavenumber to 1648 cm^−1^ [43,44,45,46,47].

Starting from SPEEK polymer, the new membranes were prepared using a casting method. The SPEEK with DS = 64% allows the preparation of a pristine membrane and several others with the incorporation of two different bisphosphonic acids as dopants in a 1.0 and 2.0 wt% load (Figure 3). The SPEEK with DS = 81% allows the formation of the membrane film; however, due to the lower stability and higher swelling properties of the membranes, the prepared pristine membrane only allowed a single determination of EIS at 40 °C before it started deteriorating [48]. This membrane presented a water uptake of 77.49% (Table 1), a very high value that can contribute to promoting its instability at high temperatures. Due to the difficulty of evaluating the polymer with this DS at temperatures above 50 °C, the study of the properties of SPEEK membranes doped with bisphosphonic acid dopants was carried out only with a SPEEK polymer matrix with a 64% sulfonation degree.

Following the devised strategy, the new membranes were prepared through the incorporation of BPs into the SPEEK polymer with a DS = 64% by a casting method using the evaporation of the solvent. Besides the preparation of a pristine membrane with no dopants, doped membranes were prepared with two different bisphosphonic acid compounds (BPs) in a 1.0 and 2.0 wt% load (Figure 3). These two BP dopants have different structural features, with **BP1** presenting two bisphosphonic groups bonded in the *para*-position of the aromatic ring, resulting in a symmetrical structure with both groups at the edges of the planar ring dopant, and **BP2** with a bisphosphonic acid group bonded at an indazole ring with two nitrogen atoms, both of which have already proved that they can improve the properties of Nafion^®^ membranes [32,38,49].

### 3.2. Membrane Characterization

**Figure 7:**
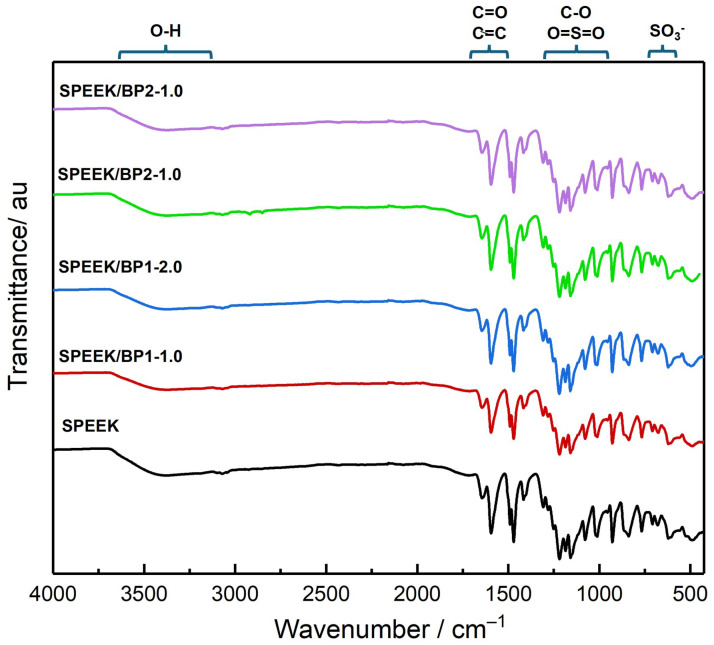
ATR-FTIR spectra of pristine and doped SPEEK membranes.

The newly prepared membranes were characterized by ATR-FTIR, and the morphological analysis of their surface was carried out by SEM. The ATR-FTIR spectra (Figure 7) present prominent bands corresponding to the sulfonic and carbonyl groups of the SPEEK. These bands, most of them with high intensity, mask the characteristic bands of dopants, with no independent bands of dopants visible at the spectra and no significant changes in the spectra due to the incorporation of the BP at these very low concentrations.

**Figure 8:**
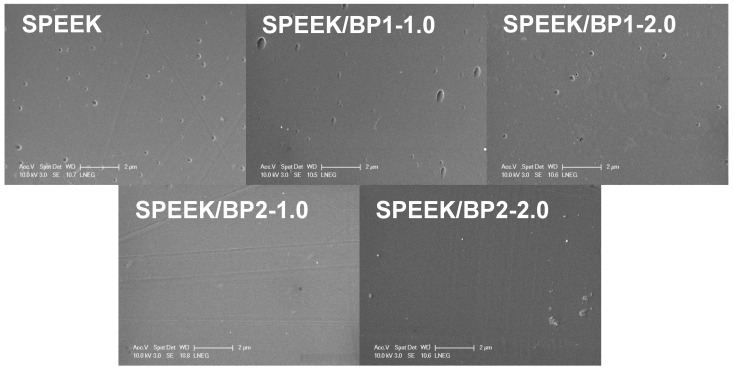
SEM surface images of prepared membranes.

The morphology analysis of the membranes afforded SEM micrographs (Figure 8) that showed a compact and dense surface with no cracks or wrinkles. The full homogeneity of their surface is only lost at pristine and **BP1** membranes, where a few small craters or cavities can be seen at the surface. A few creases or lines were observed at the surface of membranes doped with **BP2**, formed during the drying process.

Water uptake of the membranes was also measured (Table 1) by gravimetry through the determination of the weight difference between wet and dry membranes. The water uptake is very dependent on the DS, increasing with the rise of DS. In the case of the SPEEK membranes with DS = 64%, all of them presented close water uptake values, around 50%, but the pristine membrane showed the smallest water uptake (46.18%), which increased with the incorporation of the dopants to reach a 52.07% for the membrane doped with **BP2** at 2.0 wt% load. This increase suggests that acid groups of BP dopants can contribute to the retention of water molecules at the polymeric matrix, which can promote proton conduction due to their behaviour as a medium and a proton carrier throughout the membrane [50,51].

### 3.3. Proton Conductivity and Activation Energy

**Figure 9:**
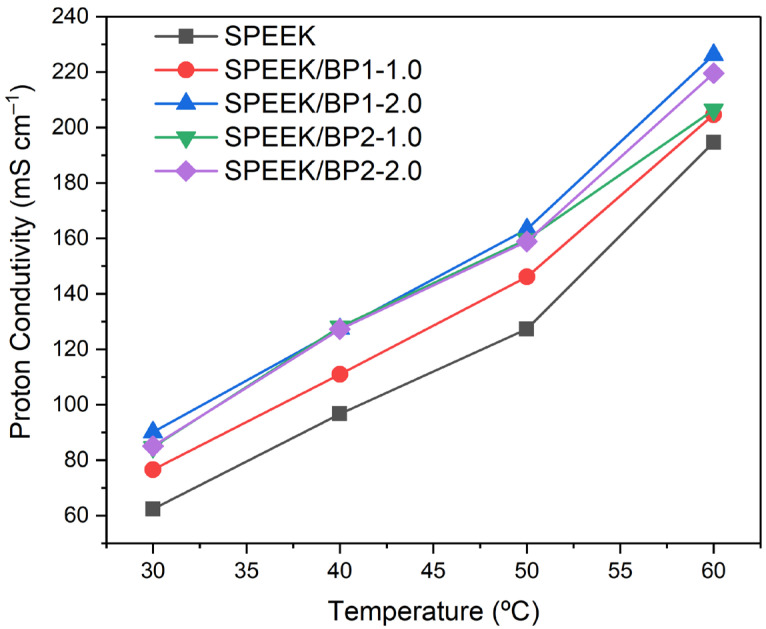
Proton conductivity of the studied membranes as a function of temperature.

The proton conduction properties of the new doped membranes were assessed by Electrochemical Impedance Spectroscopy (EIS); the pristine membrane was also evaluated for comparison. All membranes were tested at 30 °C, 40 °C, 50 °C and 60 °C temperature conditions to evaluate the proton conductivity dependency with temperature. The results showed that the proton conductivity increased with temperature, with all membranes presenting an increase in proton conductivity with the increment of temperature, with the pristine SPEEK membrane showing a 3-fold increase and the doped membranes a nearly 2.5-fold increase between 30 °C and 60 °C (Figure 9). Despite this higher increase of proton conductivity of the pristine membrane, all membranes doped with BP showed high proton conductivity at all tested temperatures.

The doped membranes showed similar behaviour in the increment of proton conductivity with the increase in temperature. In all cases, the proton conductivity increases with the rise of temperature, but their increment is similar. The membranes doped with **BP1** always showed an increment in proton conductivity with an increase in the load of the dopant. With this dopant, the best loading seems to be the 2.0 wt%, with the best proton conductivity of 226 mS cm^−1^ observed at 60 °C.

Regarding the proton conductivity of the membranes doped with **BP2**, both wt% loads showed similar results for the proton conduction at all temperatures except at 60 °C, where the membrane doped with 2.0 wt% load has a higher proton conductivity of 220 mS cm^−1^. These membranes doped with **BP2** showed proton conductivity values that are between the results observed for both **BP1** doped membranes, being closer to the doped membranes with 2.0 wt% of **BP1**.

These results seem to indicate that the structure of the BP dopant can influence the proton conductivity of the membrane, maybe through more organized incorporation at the matrix structure of the membranes, with a more symmetrical structure of **BP1** being more easily incorporated without disturbing the matrix structure of the membranes, improving the proton conduction with the increase of the wt% loading of the dopant.

Regarding all membranes, the best proton conductivities of membranes with both BP dopants are compared with other results obtained in similar conditions, already described in the literature (Table 2). The results of this study are among the best results described for SPEEK-doped membranes at similar experimental conditions, supporting the value of the implemented strategy, with BP dopants confirming their effect on promoting the proton conductivity at SPEEK membranes.

**Figure 10:**
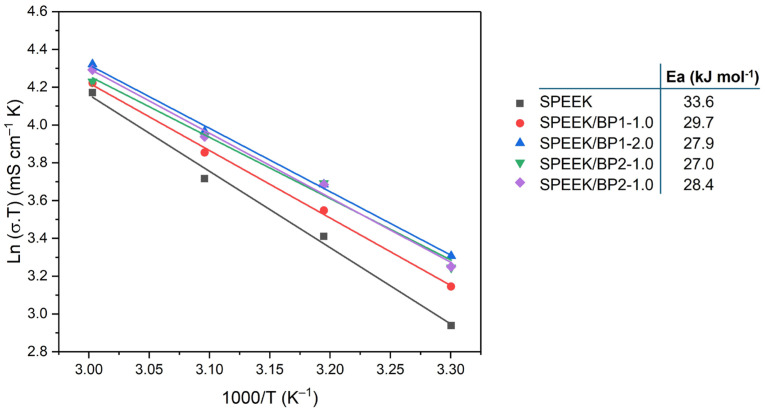
Proton conductivity (σ) Arrhenius plots of the doped membranes as a function of temperature.

The EIS results allowed the estimation of the activation energy (*E_a_*) (Figure 10) of the proton conduction in these membranes, which were calculated using the Arrhenius type temperature-dependent proton conductivity (Equation 4, adapted from [57]):

(4) $$ln\;(\sigma .T)=ln\;\sigma_{0}-\frac{E_{a}}{RT}$$

where *E_a_* is the activation energy, σ_0_ is the pre-exponential factor of the material, T is the absolute temperature (in K) and R (in J mol^−1^ K^−1^) is the ideal gas constant (used in order to have *E_a_* values in units of kJ mol^−1^).

The *E_a_* of **BP1** doped membranes showed an inverse correlation to the proton conductivity, with the lowest *E_a_* observed for the membrane doped with 2.0 wt% load. The *E_a_* of membranes doped with **BP2** is similar to the *E_a_* of the doped membrane with **BP1** at 2.0 wt% load, but for **BP2** doped membranes, the *E_a_* is smaller for the membrane with the lowest wt% load. For all doped membranes, the *E_a_* is below 30 kJ mol^−1^, below the *E_a_* of a pristine membrane that presented a value of 33.6 kJ mol^−1^; these estimated *E_a_* values are in accordance with other results already published [54,55]. These results suggested that the incorporation of BP dopants allows easier proton conduction. Either estimated *E_a_* values for pristine and doped membranes suggested that, at these experimental conditions, proton conduction occurs predominantly by a Grothuss mechanism [58,59,60].

### 3.4. Thermal Properties

**Figure 11:**
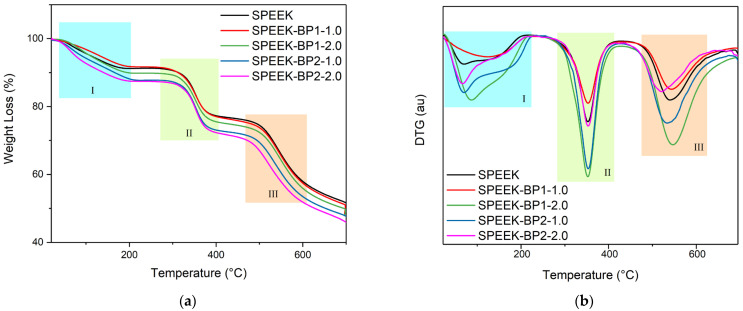
TGA (**a**) and DTG (**b**) curves of the SPEEK membranes (I, II and III—temperature ranges).

The thermal properties of the membranes of this study were submitted to thermogravimetric analysis (TGA) until 700 °C. Figure 11 shows the TGA curves of the membranes, which present three distinct weight loss temperature ranges [47,48,61]. The first temperature range starts as soon as the room temperature rises and is observed at nearly 170 °C, with their maximum centered at below 90 °C (except for membrane doped with **BP1** at 1.0 wt%, which shows a slow loss of mass, centered at 125 °C), showing a delayed loss of water by the doped membranes. This loss of weight can be attributed to the loss of water present in the matrix of the membranes. Pristine and **BP1** doped membranes lost nearly 10% of their mass at this temperature range, while **BP2** doped membranes have a loss of nearly 12% [56,62].

The second temperature range is observed between approximately 250 °C and 400 °C. These thermal losses of weight can be attributed to the thermal degradation of sulfonic acid groups and the dopants. At this temperature range, the pristine membrane showed the lower mass variation with a 14.5% loss, with the other membranes showing values from 14.9% (membrane doped with **BP1** at 1.0 wt%) to 15.6% (membrane doped with **BP2** at 2.0 wt%). The center of these temperature ranges is similar to the pristine SPEEK membrane and the other doped membranes (near 352 °C) [63].

The third temperature range is attributed to the thermal degradation of the main chain of the SPEEK membrane, with a loss of weight of higher than 24.6%. The temperature ranges start above 465 °C, and their maximum is observed between 519 °C and 546 °C [64].

The thermal analysis of these doped membranes showed that the matrix structure of SPEEK is stable until at least 450 °C. However, it starts to lose its proton conduction sulfonic acid groups and dopants at nearly 250 °C, with a higher maximum presented by the doped membranes. So, it must be noted that the incorporation of the dopants contributes to the stability of the membranes, delaying their degradation up to temperatures much higher than those required for its technological application. [65].

## 4. Conclusions

New SPEEK membranes doped with bisphosphonic acid compounds (**BP1** and **BP2**) were prepared using a casting from SPEEK polymer with a DS = 64%. The new membranes were characterized by ATR-FTIR spectroscopy, and their surface morphology was analyzed by SEM to confirm their suitability to be used as membranes on electrochemical devices.

The new membranes doped with BP dopants showed better properties than the pristine SPEEK membrane used as a reference membrane. The proton conductivity, measured by EIS, increased with the increase of the temperature. At all studied temperatures, the doped membranes showed better proton conduction than the pristine membrane. The membranes doped with **BP2** showed very small differences between them, except at 60 °C, while the membrane doped with **BP1** showed an increase in the proton conductivity with the increment of the wt% loading of the dopant. The best proton conductivity was observed at 60 °C for the membrane doped with a 2.0 wt% load of **BP1**, which exhibits a maximum proton conductivity of 226 mS cm^−1^.

The estimated *E_a_* also confirmed that the doped membranes have a lower *E_a_* than the pristine membrane. The calculated values for *E_a_* also suggested that the proton conduction at these membranes is carried out by a Grotthuss mechanism.

The TGA analysis showed that the new doped membranes have a thermally stable matrix structure that allows their use in technological applications.

Overall, the incorporation of the dopants into the new doped membranes improves their proton conductivity, sustaining the strategy of modifying membrane properties with dopants with bisphosphonic acid groups and prompting new efforts to develop new materials for electrochemical devices.

## Figures and Tables

**Table 1:** Water uptake of studied membranes.

Membranes	DS (%)	Water Uptake (%) at 25 °C
SPEEK	81	77.5 ± 1.5
SPEEK	64	46.2 ± 0.0
SPEEK/BP1-1.0	64	50.9 ± 1.7
SPEEK/BP1-2.0	64	47.2 ± 1.1
SPEEK/BP2-1.0	64	49.9 ± 3.4
SPEEK/BP2-2.0	64	52.1 ± 1.2

**Table 2:** Best proton conductivity results in this study compared with other results obtained under similar conditions.

Membranes	DS (%)	Conditions		Proton Conductivity (mS cm^−1^)	Reference
		Temperature (°C)	RH (%)		
SPEEK	64	60	100	195 *	This work
SPEEK-BP1-2.0	64	60	100	226 *	This work
SPEEK-BP2-2.0	64	60	100	220 *	This work
SPEEK/PHTS-20 ^1^	66	70	100	228	[50]
SPEEK/CN-0.5 ^2^	67	55	100	183	[52]
GO/SPEEK ^3^	70	90	100	136	[53]
S-GO/SPEEK ^4^	70	90	100	152	[53]
SPEEK	n.a.	60	100	142	[54]
SPEEK/HPW ^5^	n.a.	60	100	176	[54]
SPEEK/HPW@MSNs-0.5 ^6^	n.a	60	100	180	[54]
1%TNT-30/SPEEK ^7^	58.9	60	100	103	[55]
SPEEK/ACNT (1.5 wt%) ^8^	65	90	100	153	[43]
PI/SPEEK + HPW-20 ^9^	60.5	60	100	223	[56]
SPEEK	65	90	100	68	[51]
SPSPVG-X hybrids ^10^	65	90	100	101–122	[51]

^1^ Phosphorylated Hollow Titania Spheres. ^2^ Graphitic Carbon Nitride. ^3^ Graphene Oxide. ^4^ Sulfonated Graphene Oxide. ^5^ Phosphotungstic acid. ^6^ Phosphotungstic acid in Mesoporous Silica Nanospheres. ^7^ Titanium Dioxide Nanotubes. ^8^ Amine functionalized Carbon Nanotube. ^9^ Polyimide + Phosphotungstic acid. ^10^ Hybrid membranes of sulfonated (polyetherether ketone), Sulfonated Polyvinylidene fluoride-co-hexafluoropropylene and Graphene Oxide. * Standard deviation ≤ 1.

## Data Availability

Data is contained within the article.
